# Fine-mapping of *PmHHM*, a broad-spectrum allele from a wheat landrace conferring both seedling and adult resistance to powdery mildew

**DOI:** 10.3389/fpls.2024.1489013

**Published:** 2025-02-06

**Authors:** Bisheng Fu, Zhixin Lin, Lijuan Yan, Qiaofeng Zhang, Caiyun Liu, Jin Cai, Wei Guo, Ying Liu, Wenling Zhai, Shuangjun Gong, Feng Xu, Jizhong Wu

**Affiliations:** ^1^ Institute of Crop Germplasm and Biotechnology/Jiangsu Provincial Key Laboratory of Agrobiology, Jiangsu Academy of Agricultural Sciences, Nanjing, Jiangsu, China; ^2^ Zhongshan Biological Breeding Laboratory, Nanjing, Jiangsu, China; ^3^ Jiangsu Co-Innovation Center for Modern Production Technology of Grain Crops, Yangzhou University, Yangzhou, China; ^4^ College of Agriculture, Anhui Science and Technology University, Fengyang, Anhui, China; ^5^ School of Life Sciences, Henan University, Kaifeng, Henan, China; ^6^ Institute of Plant Protection and Soil Science, Hubei Academy of Agricultural Sciences, Wuhan, Hubei, China

**Keywords:** *Blumeria graminis*, genetic markers, resistance breeding, *Triticum aestivum* L., *PmHHM*

## Abstract

**Introduction:**

Common wheat is a leading global food crop that impacts food security. Wheat powdery mildew (PM), caused by *Blumeria graminis* f. sp. *tritici* (*Bgt*), poses a significant threat to grain yield and flour quality. The identification and utilization of broad-spectrum resistance genes against PM are essential for effective disease control.

**Methods:**

The resistance spectrum test during the seedling stage and the identification of resistance during the adult stage were conducted to evaluate the wheat landrace Honghuamai (HHM). Five segregating populations were investigated to assess the inheritance of PM resistance in HHM. To map its PM resitance gene, bulked segregant analysis, molecular mapping and comparative genomic analysis were also used in the present study.

**Results:**

HHM shows remarkable adult resistance in the field and is nearly immune to all 25 *Bgt* isolates used in seedling tests, making it an excellent source of PM resistance. PM resistance in HHM was determined by a single dominant gene, temporarily named *PmHHM*. It was then fine-mapped to an interval with a genetic distance of 0.0031 cM and a physical distance of 187.4 kb on chromosome 4AL of the Chinese Spring reference sequence v.2.1. Four genes were identified in the target region, three of which encode nucleotide-binding leucine-rich repeat (NLR) proteins. Comparative genomic analysis revealed presence/absence variations (PAVs) of the *PmHHM* locus among common wheat varieties.

**Discussion:**

These closely linked molecular markers will not only benefit the cloning of the gene underlying *PmHHM* but also facilitate the efficient utilization of the gene in breeding programs.

## Introduction

Common wheat (*Triticum aestivum* L.) is an important global food crop, and its stable production contributes significantly to global food security. Wheat powdery mildew (PM), caused by *Blumeria graminis* f. sp. *tritici* (*Bgt*), is a major fungal disease affecting wheat production (Juroszek and von Tiedemann, 2012; Singh et al., 2016). Although chemical and biological methods can be used to reduce the impacts of this disease, the utilization of effective disease resistance genes remains the most economical, efficient, and environmentally friendly strategy.

Since the naming of the first PM resistance gene, *Pm1* (Pugsley and Carter, 1953), over 200 resistance genes/alleles and quantitative resistance loci (or quantitative trait loci (QTL)) have been reported (McIntosh et al., 2020; Li et al., 2024a). At present, at least 26 genes associated with resistance to PM have been cloned. The proteins produced by these genes are categorized into three distinct groups: nucleotide-binding leucine-rich repeat (NLR) proteins, kinase proteins, and transporter proteins. The following genes were categorized as kinase proteins: *Pm4* (Sánchez-Martín et al., 2021), *Pm13* (Li et al., 2024b; He et al., 2024a), *Pm24* (Lu et al., 2020), *Pm36* (Li et al., 2024c), *Pm57* (Zhao et al., 2024), and *WTK4* (Gaurav et al., 2022). In contrast, the genes *Pm38*/*Yr18*/*Lr34*/*Sr57* (Krattinger et al., 2009) and *Pm46*/*Yr46*/*Lr67*/*Sr55* (Moore et al., 2015) were classified as transporter proteins. The remaining genes belong to the NLR category, which includes *Pm1a* (Hewitt et al., 2021), *Pm2a* (Sánchez-Martín et al., 2016), *Pm3b*/*Pm8*/*Pm17* (Yahiaoui et al., 2004; Hurni et al., 2013; Singh et al., 2018), *Pm5e* (Xie et al., 2020), *Pm21*/*Pm12* (He et al., 2018; Xing et al., 2018; Zhu et al., 2023), *Pm41* (Li et al., 2020), *Pm55* (Lu et al., 2024), *Pm57* (Zhao et al., 2024), *Pm60*/*MlIW172*/*MlWE18* (Zou et al., 2018; Wu et al., 2021a, 2022), *Pm69* (Li et al., 2024a), *PmTR1*/*PmTR3* (Han et al., 2024), *PmAeu1* (He et al., 2024b), and *Pm6Sl* (Ma et al., 2024). Most of these genes can be easily overcome by existing or newly generated virulent isolates, such as *Pm1a*, *Pm3c*, *Pm5a*, and *Pm8* in China (Zeng et al., 2014). Therefore, the continuous exploration and utilization of PM resistance genes are important for wheat geneticists and breeders.

Wheat landraces are important genetic resources for wheat improvement. Many PM genes have been identified from landraces or their derivatives. They include *Pm2c* (Xu et al., 2015), *Pm5d* (Hsam et al., 2001), *Pm5e* (Xie et al., 2020), *Pm24* (Lu et al., 2020), *Pm47* (Xiao et al., 2013), *Pm59* (Tan et al., 2018), *Pm61* (Sun et al., 2018), *Pm63* (Tan et al., 2019), and those temporarily named *pmX* (Fu et al., 2013), *pmHYM* (Fu et al., 2017), *PmHSM* (Fu et al., 2024), *PmDGM* (Wu et al., 2021b), *PmHHXM* (Xue et al., 2021), and *PmXNM* (Xue et al., 2024). Although landraces carry invaluable favorable genes, the majority of such genotypes have poor agronomic traits, especially lower yields (Zeven, 1998). In order to eliminate unfavorable linkage drag related to the target loci, fine-mapping of targeted loci and development of diagnostic molecular markers are of great significance to accelerate the utilization of these genes.

Plants have developed two main types of defenses to prevent pathogen infections: one is quantitative non-race-specific, and the other is qualitative race-specific disease resistance (Michel et al., 2023). Non-race-specific resistance genes typically provide partial, quantitative resistance against all pathogen races, while race-specific resistance offers complete protection against specific races and is controlled by single resistance genes (Sánchez-Martín and Keller, 2021). Certain genes, such as *Pm21*, exhibit high levels of resistance against most *Bgt* isolates and are recognized for conferring broad-spectrum resistance (He et al., 2018; Xing et al., 2018; Liu et al., 2021). Therefore, the discovery of such resistance genes holds great significance for wheat breeding against PM. Honghuamai (HHM) is a Sichuan wheat landrace with excellent PM resistance, providing high levels of resistance to 25 representative *Bgt* isolates in China. To determine the genetic basis of its resistance and lay a foundation for its utilization, we carried out this study with the following objectives: (1) genetic analysis of PM resistance in HHM at the seedling and adult stages, (2) fine-mapping of *PmHHM*, (3) identification of candidate genes for *PmHHM*, and (4) assessment of the relationship between *PmHHM* and other reported PM genes and loci.

## Materials and methods

### Plant materials and *Bgt* isolates

**Figure 1:**
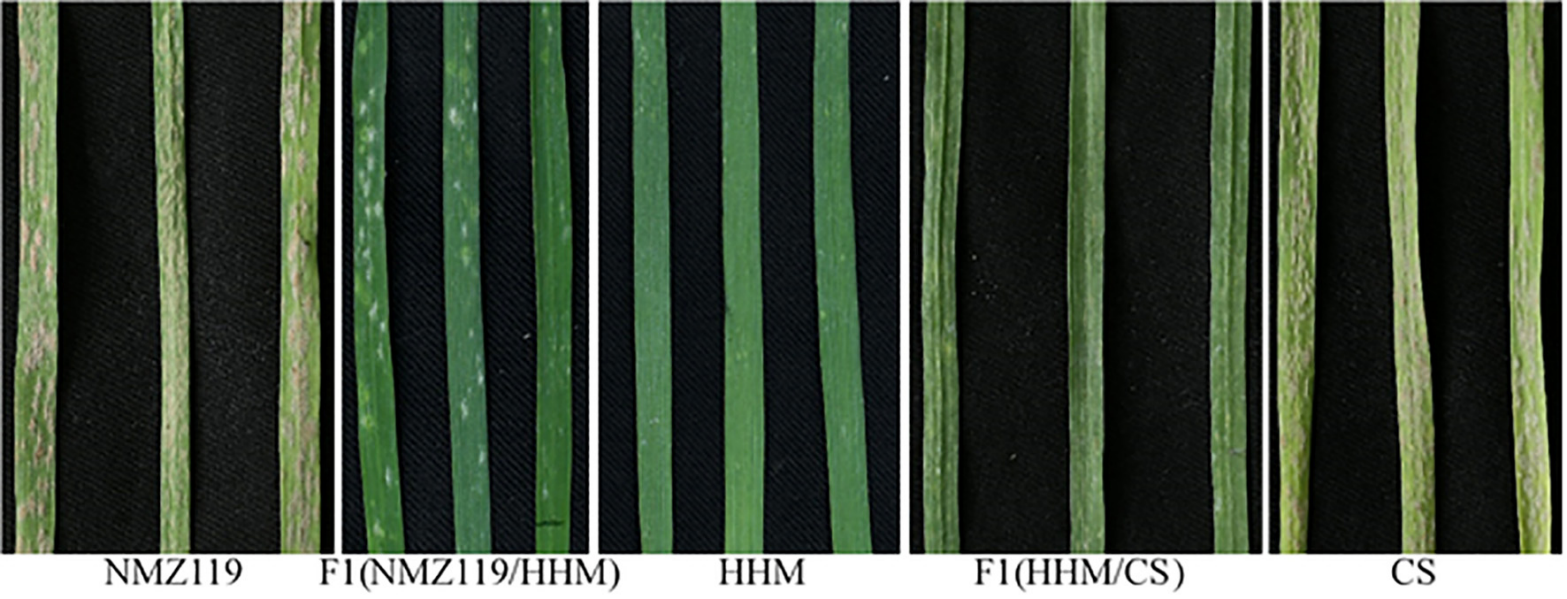
Powdery mildew responses of susceptible parent NMZ119 (IT 4), F1 NMZ119/HHM (IT 1), resistant parent HHM (IT 0–1), F1 HHM/CS (IT 1), and susceptible parent CS (IT 4).

HHM was crossed with PM-susceptible wheat varieties NingmaiZi119 (NMZ119, **Figure 1**) and Chinese Spring (CS, **Figure 1**). Populations of F_1_, F_2_, F_3_, BC_2_F_2_, and BC_3_F_1_ were utilized for the genetic analysis of PM resistance in HHM. In addition to the susceptible parents, Heshangmai (HSM), Honghuaxiaomai (HHXM), Chancellor (CC), and Zhongzuo (ZZ) were also used as controls. The crosses HHM/HSM and HHM/HHXM were used for testing allelism. All of the genotypes used in this study were sourced from the crop germplasm bank of the Jiangsu Academy of Agriculture.

A total of 25 *Bgt* isolates collected from various regions of China were used in this study. Among them, *Bgt* isolate B03 was provided by Professor Hongxing Xu from the School of Life Sciences at Henan University and is the most prevalent isolate in the Nanjing area. Additionally, 18 isolates were obtained from a collection maintained at Hubei Academy of Agricultural Sciences in Wuhan (Zeng et al., 2014), while six isolates were sourced from Dr. Hongjie Li’s laboratory at the Chinese Academy of Agricultural Sciences in Beijing. Detailed information on the *Bgt* isolates is provided in **Supplementary Table S1.**

### Resistance evaluation

Assessments of PM response were conducted following the procedure outlined in Zeng et al. (2014). The test materials, resistant parent HHM and susceptible control Sumai 3 (S3), were randomly sown in 50-hole trays (5 cm × 5 cm) for seedling response assays. The trays were placed in an artificial climate chamber. The settings for the climate chamber were as follows: 14-h day/10-h night cycle, 22°C day/18°C night temperature, and 50% to 60% relative humidity.

At the one-leaf stage, the seedlings were dust-inoculated using sporulating plants infected with the *Bgt* isolate B03. The relative humidity of the climate chamber was increased to 80%–100% following the inoculation. The disease responses of HHM, HSM, and HHXM to various *Bgt* isolates were assessed using 25 isolates, with CC and ZZ serving as susceptible controls. Disease severity was evaluated when the susceptible controls S3, CS, and NMZ119 displayed full sporulation. Scoring of infection types (ITs) was carried out by utilizing a scale ranging from 0 to 4 (Fu et al., 2017), comprising six levels: 0, 0;, 1, 2, 3, and 4. The results were classified based on ITs, with ITs 0 to 2 categorized as resistant and ITs 3 to 4 labeled as susceptible.

To investigate the inheritance of adult plant PM resistance in HHM at the adult stage, in 2021 we inoculated and phenotyped 1,352 NMZ119/HHM F_2_ plants and 820 HHM/CS F_2_ plants that were susceptible at the seeding stage. Following this, we inoculated and phenotyped the BC_2_F_2_ and BC_3_F_1_ populations of NMZ119/HHM in 2023. All of these plants were grown in an isolated net house that contained no other wheat or *Bgt* isolates, thereby ensuring there was no contamination from any other *Bgt* isolates. To ensure uniform infection, S3 was planted around each F_2_ plants. In mid-February, S3 was inoculated with *Bgt* isolate B03, and the disease responses were recorded during the flowering period in April when S3 was fully susceptible (IT = 8 or 9), using the 11-point scale of Sheng and Duan (1991)—that is, ITs 0, 0;, 1, 2, 3, 4, 5, 6, 7, 8, and 9. The entire plant is divided into nine equal sections from the bottom to the top, and the resistance level is determined based on the severity of diseased leaves (or spikes) at different heights. The levels are defined as follows: 0, immune—no disease spots present on the entire plant; 0;, near immune—leaves show dead spots but have no disease spots; 1, high resistance—a few lesions are present on the leaves in the first segment (lesions account for less than 2% of the total leaf area); 2, high resistance—a few lesions appear on the leaves in the second segment, with mild symptoms in the first segment (lesions account for 3%–10% of the leaf area); 3, moderate resistance—mild symptoms occur in the third leaf segment, moderate disease in the second leaf segment (lesions account for 11%–25% of the leaf area), and severe disease in the first leaf segment (lesions account for more than 25% of the leaf area); 4, moderate resistance—mild symptoms in the fourth leaf segment, with moderate to severe symptoms in the third segment and below; 5, moderate infection—mild symptoms in the fifth leaf segment, with moderate to severe symptoms in the fourth segment and below; 6, moderate infection—mild symptoms in the sixth leaf segment, with moderate to severe symptoms in the fifth segment and below; 7, highly sensitive—mild symptoms in the seventh leaf segment, with moderate to severe symptoms in the sixth segment and below. Occasionally, the flag leaves may show a few disease spots; 8, highly sensitive–mild or moderate disease affects the flag leaf, with moderate to severe disease present in the leaves below the flag leaf; 9, extremely sensitive—the entire leaf is severely affected, and the ear may show varying degrees of damage. ITs 0–4 were regarded as resistant, while ITs 5–9 were classified as susceptible.

Based on the identification criteria for both seedling and adult stages outlined above, the identified population is categorized into two groups: resistant and susceptible. By analyzing the ratio of resistant plants to susceptible plants, we can infer the potential genetic ratio. Chi-square tests were employed to determine whether the observed segregation ratios aligned with the expected ratios.

### Bulked segregant analysis and mapping of *PmHHM*

The procedure for DNA extraction used in this study was that outlined by Fu et al. (2017). F_3_ families from the cross NMZ119/HHM were genotyped using mixtures of equal amounts of DNA from 10 randomly selected homozygous resistant families (the resistance pool) and equal amounts of DNA from 10 randomly selected homozygous susceptible families (the susceptibility pool). A 55K SNP chip was utilized to analyze these DNA samples along with DNA from the parents. This process aimed to detect SNP markers displaying consistent polymorphisms between the parental samples and between the two pools. When *PmHHM* was located within a specific physical interval, primers were designed to amplify specific regions within the interval based on CS RefSeq v2.1. Sequence variations between HHM and CS were utilized in developing various molecular markers, including SSR, KASP, CAPS, dCAPS, and SNP markers. The markers were developed on PrimerServer of WheatOmics 1.0 (http://202.194.139.32/PrimerServer/). PCR amplifications and detection of markers were carried out following the procedures detailed by Fu et al. (2017). For the detection of KASP markers, standard FAM tail (5′-GAAGGTGACCAAGTTCATGCT-3′) and HEX tail (5′-GAAGGTCGGAGTCAACGGATT-3′) sequences were incorporated at the 5′-ends of HHM allele (A)- and CS allele (B)-specific primers, respectively. The KASP marker reactions were carried out using a Hydrocycler-16 Water Bath Thermocycler (LGC Genomics, Hoddesdon, Herts, UK), with fluorescence detection performed utilizing a PHERAstar high-end microplate reader (LGC Genomics).

To conduct fine-mapping of *PmHHM*, markers flanking the gene from the initial mapping were employed to screen DNA from homozygous susceptible F_2_ plants in the NMZ119/HHM and HHM/CS populations. Genotypic analysis of recombinants was carried out using newly developed SSR markers, dCAPS markers, KASP markers, and SNP markers to construct a saturated genetic map of the *PmHHM* region. To confirm the homozygous susceptibility of the recombinants, self-pollinated seeds (20–30 seeds) were utilized to assess PM resistance. Fine-mapping of *PmHHM* was accomplished by comparing the genotype–phenotype relationships of the recombinants. The sequences for all markers used in this study are listed in **Supplementary Table S2**.

### Physical mapping of *PmHHM* and recombination rate estimation

To anchor *PmHHM* on the CS genome, sequences of markers linked to the gene were aligned with CS RefSeq. v2.1. Markers linked to *Pm61* (Sun et al., 2018), *PmHSM* (Fu et al., 2024), *MlIW30* (Geng et al., 2016), *QPm*.*tut*-*4A* (Janáková et al., 2019), *PmPBDH* (Liang et al., 2022), *MlNFS10* (Yin et al., 2021), *PmHHXM* (Xue et al., 2021), and *PmXNM* (Xue et al., 2024) were also aligned to determine their physical locations in the CS genome. The recombination rate was calculated using the ratio of genetic distance (cM) to physical distance (Mb).

### Comparative genomic analysis and prediction of candidate genes in the *PmHHM* region

The genomic sequence of the *PmHHM* region in CS (chromosome arm 4AL, 740–754 Mb) and that of 13 other sequenced wheat cultivars were obtained from the WheatOmics 1.0 (http://202.194.139.32). The CS sequence was compared with the corresponding sequences of the other cultivars using the MUMmer 3.0 package (Kurtz et al., 2004). The alignments were filtered based on identity (≥85%) and length (≥5,000 bp) criteria and visualized using the MUMmer 3.0 package. The candidate genes for *PmHHM* were predicted according to JBrowse in WheatOmics 1.0 (http://202.194.139.32/jbrowse-1.12.3-release/?data=Chinese_Spring2.1).

### Construction of genetic and physical maps

Chi-square (*χ* ^2^) tests were conducted to assess genetic ratios. The genetic distances in the preliminary map were calculated using MAPmarker 3.0, with a LOD value set at 3.0. The recombination rate of the fine-map was calculated using the formula *r* = (2f1 + f2)/2*N*, where f1 represents the number of homozygous recombinants, f2 is the number of heterozygous recombinants, *N* is the number of screened individual plants, and the recombination value was converted to map distance using the formula *d* = {ln (1 + 2r)/(1 - 2r)}/4. The resulting genetic and physical maps were visualized using MapDrawV2.1 (Liu and Meng, 2003).

## Results

### Inheritance of PM resistance in HHM

HHM showed high resistance to each of the 25 *Bgt* isolates (**Table 1**; **Figure 1**), indicating that it has highly effective, broad-spectrum resistance at the seedling stage. The F_1_ plants from both NMZ119/HHM (IT = 1, **Figure 1**) and HHM/CS (IT = 1, **Figure 1**) plants were resistant, indicating that the resistance is dominant. The segregation data (**Table 2**) showed clear evidence for segregation of a single gene in both crosses. The resistance allele in HHM is temporarily named *PmHHM.*

**Table 1:** Reaction patterns of *PmHHM*, *PmHSM*, and *PmHHXM* to 25 *Blumeria graminis* f. sp. *tritici* (*Bgt*) isolates at the seedling stages.

Accessions (gene)	*Bgt* isolates																								
	1-19	11-99	12-3	14-32	15-9-4	15-17	21-2	37-38-10	46-30	48-18	48-28	NJ-16	WH-11	96224	HB-24(SWQ)	E21-4	39-19	46-25	F02	A44	F17	F26	E20	F24	B03
HHM (*PmHHM*)	1	0	0	0	0	0	0	1	0	1	1	1	0	0	0	0	1	1	0	0	0	0	0	0	1
HSM (*PmHSM*)	1	0	0	0	0	0	0	1	0	1	1	1	4	0	0	0	4	1	0	0	0	0	0	0	1
HHXM (*PmHHXM*)	0	0	0	0	0	0	0	0	0	1	0	1	0	0	1	0	0	4	0	0	0	0	0	0	0
NMZ119 (unknown)	0	1	2	4	2	3	0	0	4	4	4	4	4	4	2	4	4	1	0	0	2	2	2	2	4
CC (susceptible control)	4	4	4	4	4	4	4	4	4	4	4	4	4	4	4	4	4	4	–	–	–	–	–	–	–
ZZ (susceptible control)	–	–	–	–	–	–	–	–	–	–	–	–	–	–	–	–	–	–	4	4	4	4	4	4	4
S3 (susceptible control)	–	–	–	–	–	–	–	–	–	–	–	–	–	–	–	–	–	–	–	–	–	–	–	–	4

HHM, Honghuamai; HSM, Heshangmai; HHXM, Honghuaxiaomai, NMZ119, Ningmaizi119; CC, Chancellor; ZZ, Zhongzuo; S3, Sumai 3; -, lack of experimental data; these results repeat three times.

**Table 2:** Inheritance of resistance to powdery mildew (*Bgt* isolate B03) at seedling stages in HHM.

Cross	F_1_	F_2_				F_2:3_		
		R:S	Predicted ratio	*χ* ^2^	*P*	RR: Rr:rr	*χ* ^2^_1:2:1_	*P*
NMZ119/HHM	R	3,812:1,352	3:1	3.78	0.052	36:78:40	0.23	0.89
HHM/NMZ119	R	115:41	3:1	0.077	0.78	36:79:41	0.35	0.84
HHM/CS	R	2,465:820	3:1	0.00091	0.98	84:171:100	1.92	0.38
NMZ119*^3^/HHM (BC_2_F_2_)	–	98:37	3:1	0.30	0.58			
NMZ119*^4^/HHM (BC_3_F_1_)	–	46:50	1:1	0.094	0.76			
HHM/HHXM	R	1,801:0	15:1	119.00	1.05 × 10^-27^			
HHM/HSM	R	1,204:0	13:3	276.62	4.10 × 10^-62^			

HHM, Honghuamai; NMZ119, Ningmaizi119; CS, Chinese Spring; *χ* ^2^, chi-square; *P*, probability of conforming to the assumed model; R, resistance; S, susceptible; RR, homozygous resistant; Rr, segregating; rr, homozygous susceptible.

HHM was also resistant to PM at the adult stage (IT 1–2). We identified 1,352 susceptible NMZ119/HHM F_2_ plants and 820 susceptible HHM/CS F_2_ plants that exhibited susceptibility (IT = 7–8) during the adult stage, consistent with the results of seedling evaluation. Based on the genomic composition of these plants and a sufficiently large population sample, we concluded that *PmHHM* is responsible for mediating adult resistance. To further validate the results, phenotyping of adult plants from the two backcross populations was conducted, and the results completely agreed with those obtained at the seedling stage (**Table 2**). The bimodal distribution observed (**Supplementary Figure S1**) again confirmed that *PmHHM* not only mediates resistance to PM at the seedling stage but also confers resistance at the adult stage.

### Chromosome location and preliminary mapping of *PmHHM*

Analyzing HHM, NMZ119, and the resistant and susceptible pools using the wheat 55K SNP array identified 1,194 polymorphic SNPs distributed across all the 21 wheat chromosomes (**Supplementary Figure S2a**). Among the SNPs, 231 were found on chromosome 4A (19.35%), 212 on chromosome 6D (17.76%), and 192 on chromosome 5A (16.08%). Further analysis revealed enrichment in the 700–750-Mb region on the chromosome arm 4AL (**Supplementary Figure S2b**).

**Figure 2:**
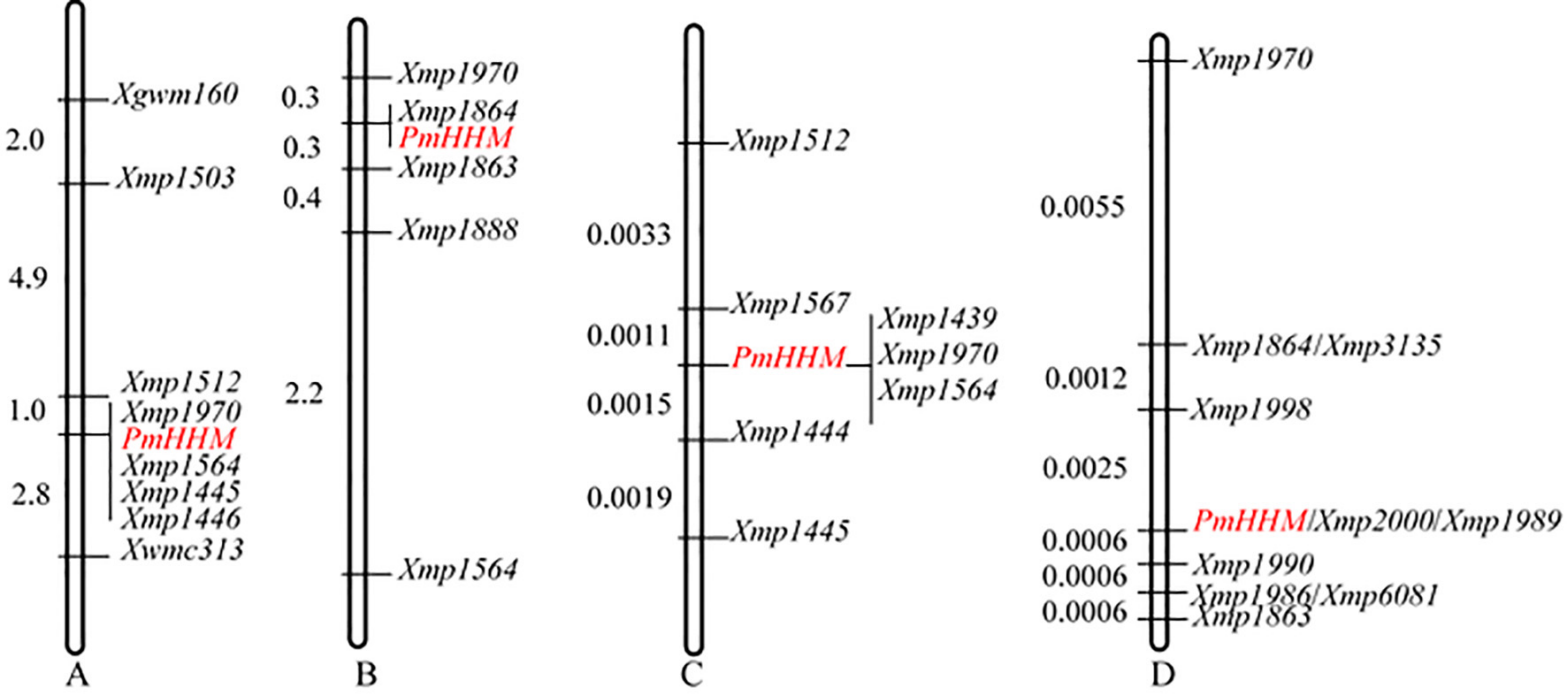
Genetic mapping of *PmHHM*. **(A)** A preliminary genetic map constructed using 310 plants, including 156 F_2_ plants from HHM/NMZ119 and 154 F_2_ plants from NMZ119/HHM; **(B)** a preliminary genetic map constructed using 355 F_2_ plants from HHM/CS; **(C)** a detailed genetic map constructed using 1,352 susceptible F_2_ plants from NMZ119/HHM; **(D)** a detailed genetic map constructed using 820 susceptible F_2_ plants from HHM/CS.

Further analysis of the 700–750-Mb region on chromosome arm 4AL showed that *Xgwm160* and *Xwmc313* were polymorphic between HHM and NMZ119 as well as between the R and the S pools. However, they were not polymorphic between HHM and CS. Therefore, these two markers were genotyped in 154 families from the NMZ119/HHM cross and 156 families from the reciprocal HHM/NMZ119 cross. *PmHHM* was mapped between *Xgwm160* and *Xwmc313*. For fine-mapping *PmHHM*, 15 newly developed SSR markers based on CS RefSeq v2.1 were analyzed. Markers *Xmp1503*, *Xmp1512*, *Xmp1445*, and *Xmp1446* were polymorphic among HHM, NMZ119, and two pools (**Supplementary Figure S3**), whereas *Xmp1888*, *Xmp1863*, *Xmp1864*, *Xmp1970*, and *Xmp1564* were polymorphic among HHM, NMZ119, CS, and their corresponding pools (**Supplementary Figure S3**). *PmHHM* was further mapped within the marker intervals of *Xmp1512*–*Xwmc313* (**Figure 2A**) and *Xmp1970*–*Xmp1863* (**Figure 2B**) between these two populations, respectively.

When different populations were used for mapping, clear differences in the recombination rates were noticed among them. To investigate this phenomenon, we conducted a detailed analysis of the recombination rates in gene regions, distal-telomere regions, and proximal-telomere regions (**Table 3**). Our results showed that (1) the recombination rates of the NMZ119 population were lower than those of the CS population and (2) the recombination rate of the targeted region was lower than those on either side in the NMZ119 population.

**Table 3:** Recombination rates of molecular markers in the *PmHHM* region.

Distal-telomere region		*PmHHM* region		Proximal-telomere region		Average recombination rate (cM/Mb)	Population
Position	Recombination rate (cM/Mb)	Position	Recombination rate (cM/Mb)	Position	Recombination rate (cM/Mb)		
*Xgwm160*–*Xmp1512*	0.53	*Xmp1512*–*Xmp1445*	0.15	*Xmp1445*–*Xwmc313*	3.73	0.53	310 F_2_ plant derived from HHM and NMZ119
*Xmp1512*–*Xmp1567*	0.0024	*Xmp1567*–*Xmp1444*	0.00056	*Xmp1444*–*Xmp1445*	0.0029	0.0012	1,352 susceptible F_2_ plants derived from NMZ119/HHM
*Xmp1970*–*Xmp1864*	0.46	*Xmp1864*–*Xmp1863*	0.65	*Xmp1863*–*Xmp1564*	1.58	1.19	355 F_2_ plants derived from HHM/CS
*Xmp1970*–*Xmp1998*	0.0099	*Xmp1998*–*Xmp1990*	0.016	*Xmp1990*–*Xmp1863*	0.0048	0.0098	820 susceptible F_2_ plants derived from HHM/CS

cM, centimorgans; Mb, Megabase; HHM, Honghuamai; NMZ119, Ningmaizi 119; CS, Chinese Spring.

### Fine-mapping of *PmHHM*

**Figure 3:**
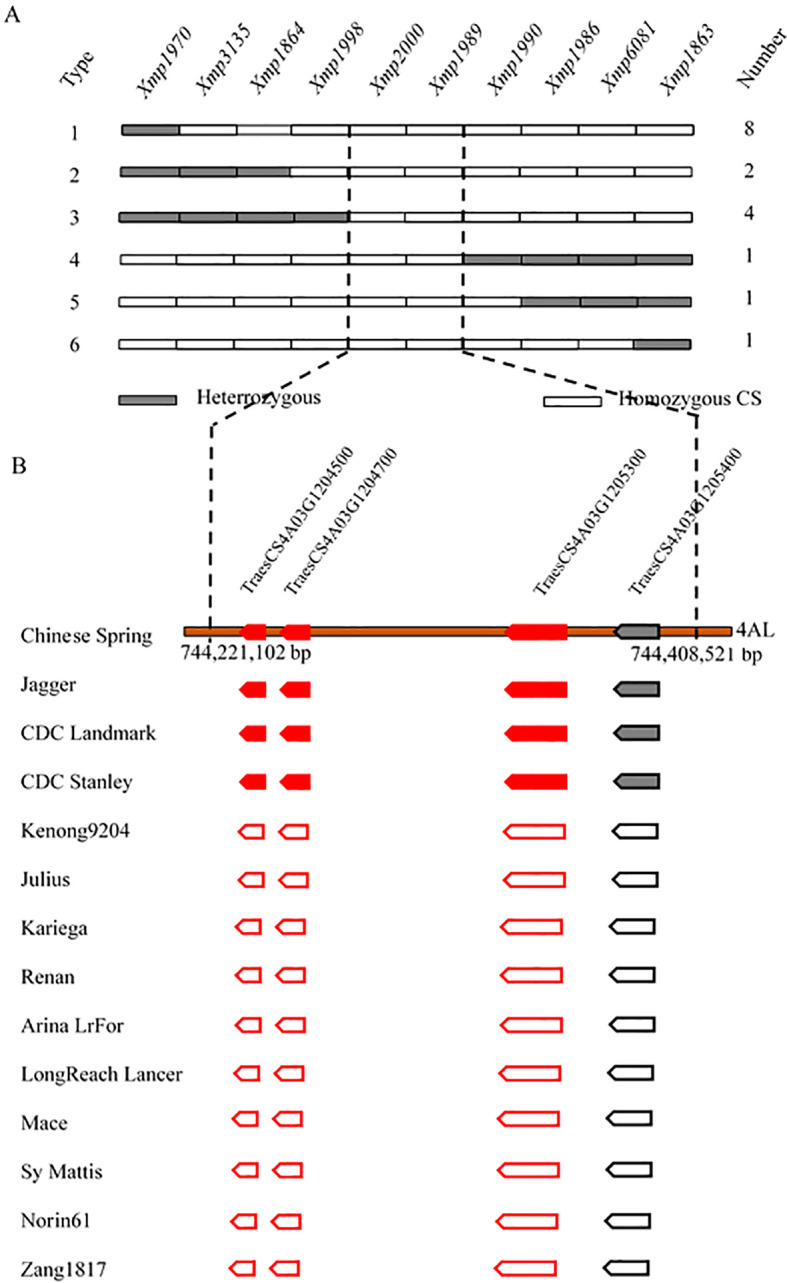
Fine-mapping and candidate gene analysis of *PmHHM*. **(A)** Graphical genotype of 17 homozygous susceptible recombinants identified between markers *Xmp1970* and *Xmp1863* from 820 susceptible F_2_ plants from the HHM/CS cross. Gray and empty rectangles represent heterozygous genotypes and homozygous genotypes, respectively. **(B)** Presence/absence variations of four high-confidence genes annotated within the 187.4-kb target region of Chinese Spring in 13 other sequenced common wheat cultivars. Gene deletions are depicted as empty pentagons. The annotated genes associated with disease resistance are highlighted as red solid boxes and bold font.

The two molecular markers flanking the targeted locus (*Xmp1512* and *Xmp1445*) were used to analyze 1,352 susceptible plants from the NMZ119/HHM population. *Xmp1512* identified 12 heterozygous lines, and *Xmp1445* identified nine (**Supplementary Table S3**). Analyzing 820 susceptible lines from the population of HHM/CS using *Xmp1970* and *Xmp1863* identified 14 and 3 heterozygous lines, respectively (**Figure 3A**). Further saturation of the genetic map by genotyping existing and newly developed markers against the NMZ119/HHM population narrowed down the location of *PmHHM* in the NMZ119/HHM population to a 0.0026-cM region, with a physical distance of 4.46 Mb between markers *Xmp1567* and *Xmp1444* (**Figure 2C**). In the HHM/CS population, *PmHHM* was mapped to a 0.0031-cM genetic interval of 187.4 kb (CS RefSeq v2.1) between *Xmp1998* and *Xmp1990* (**Figures 2D**, **3B**). The lower recombination rate in the NMZ119 population and the higher recombination rate in the CS population were also apparent in the fine-mapping analysis (**Table 3**).

### The relationship of *PmHHM* and other PM resistance genes located on 4AL

Several genes conferring PM resistance were found to be located in the region of *PmHHM*. They include *Pm61*, *PmHSM*, *MlIW30*, *QPm*.*tut*-*4A*, *PmPBDH*, *MlNFS10*, *PmHHXM*, and *PmXNM*. However, we could only be able to obtain seeds of the HSM and HHXM from the eight wheat differentials for resistance spectral analysis at the seeding stage and for allelic testing. As shown in **Table 1**, HHM was resistant to all 25 *Bgt* isolates, whereas HSM was susceptible to two of them (WH-11 and 39-19), and HHXM was susceptible to only one of them (46-25). Assuming that a single resistance gene was responsible for PM resistance in each of these lines, these results indicated that these genotypes possess different genes. To ascertain the resistance spectrum of *PmHHM*, 10 randomly chosen homozygous resistant F_2:3_ lines from NMZ119/HHM were inoculated with the 25 *Bgt* isolates. All 10 F_2:3_ lines were homogeneously resistant to all of the 25 isolates, indicating that *PmHHM* conferred a broad-spectrum resistance to this disease.

**Figure 4:**
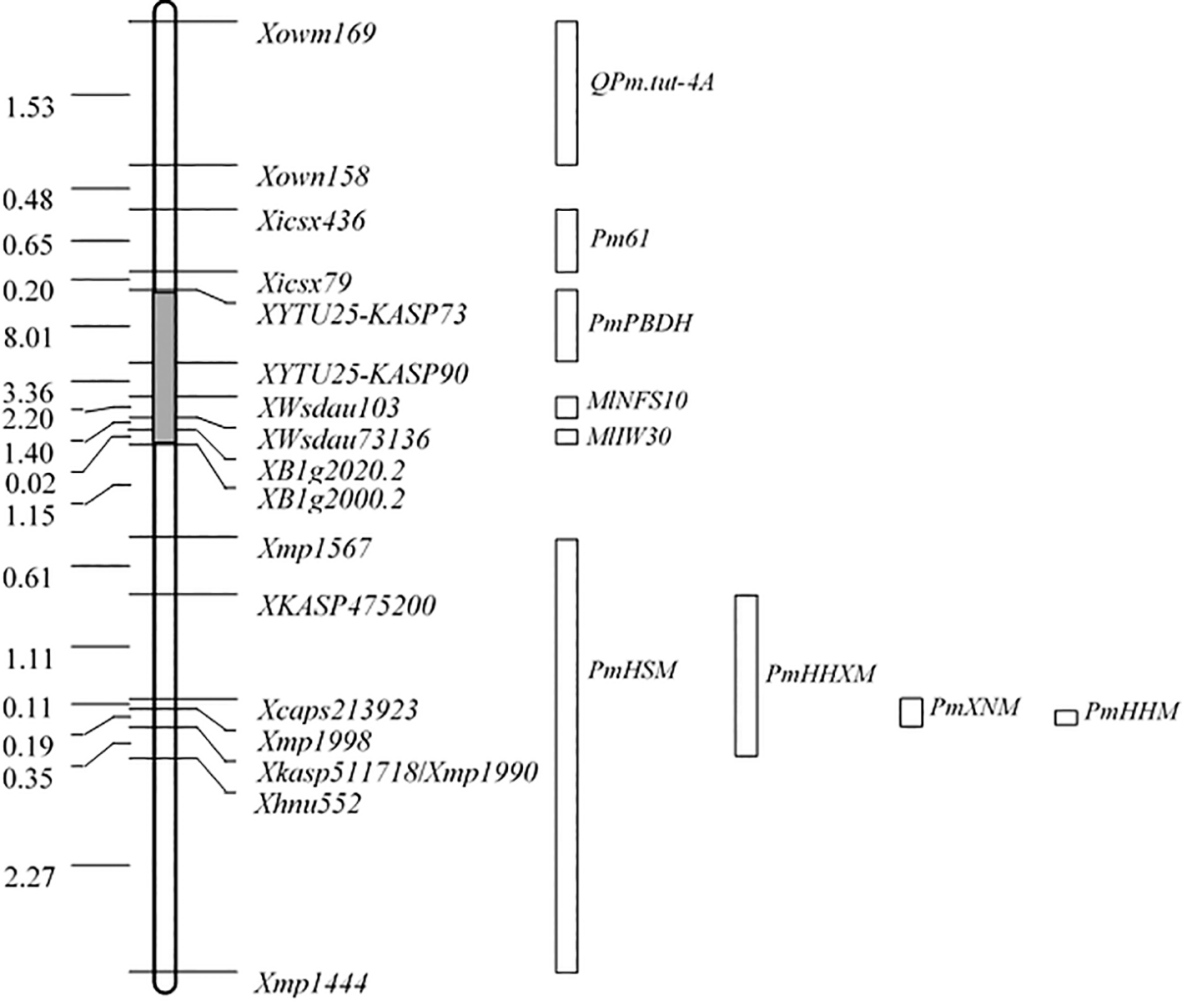
Comparison of the physical positions of *QPm*.*tut*-*4A*, *Pm61*, *PmPBDH*, *MLIW30*, *PmHHXM*, *MlNFS10*, *MlIW30*, *PmHSM*, *PmHHXM*, *PmXNM*, and *PmHHM* on chromosome arm 4AL based on sequences of CS RefSeq v2.1. Locus names and corresponding physical distances (Mb) are displayed on the left and right sides of the map, respectively. The gray region on the map is not drawn to scale. The boxes at the right denote the physical positions of nine genes from Xue et al. (2024) and Fu et al. (2024).

A total of 13 F_1_ and 1,801 F_2_ plants from the population of HHM/HHXM and 10 F_1_ and 1,204 F_2_ plants from HHM/HSM were all resistant to isolate B03 (**Table 2**). Under the conditions of a chi-square test with a *P*-value of 0.05, the maximum genetic distances from *PmHHM* to *PmHHXM* and from *PmHHM* to *PmHSM* are 0.0043 and 0.0064 cM, respectively, indicating that *PmHHM*, *PmHHXM*, and *PmHSM* are likely allelic or closely linked to each other. Although we did not obtain the other six materials, we anchored all nine genes on the CS genome and constructed physical location maps of these genes (**Figure 4**). Based on the physical location of the genes on the chromosome, we determined that *PmHHM* differs from *Pm61*, *PmPBDH*, *MlNFS10*, *MlIW30*, and *QPm.tut-4A* and is allelic or closely linked to *PmHHXM*, *PmXNM*, and *PmHSM*.

### Comparative genomic analysis and candidate gene prediction

**Figure 5:**
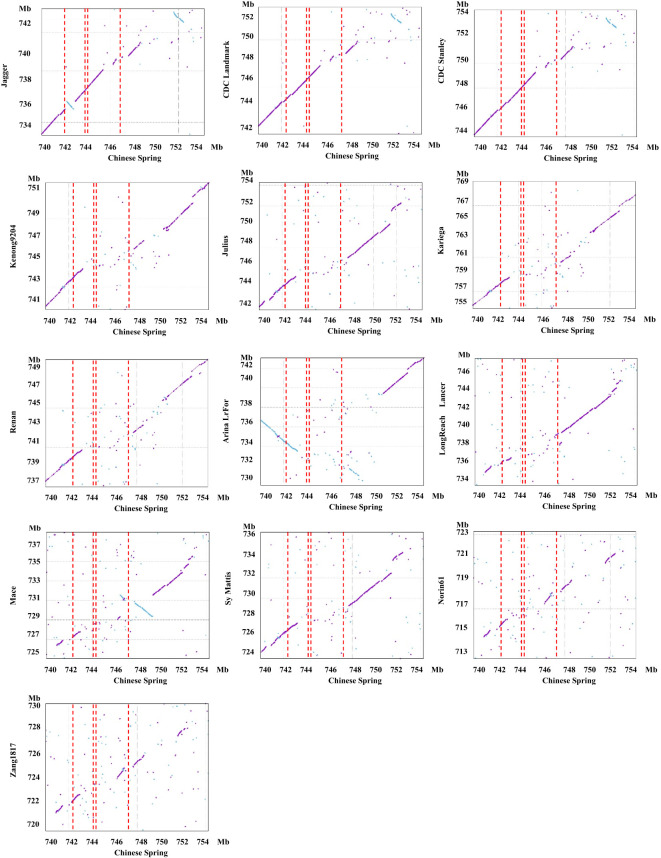
Synteny analysis of the targeted 740–754-Mb region on chromosome arm 4AL between Chinese Spring and 13 other sequenced common wheat cultivars. The red dashed rectangle defines the interval harboring *PmHHM*. The first and fourth red dashed lines represent the physical distance of 4.46 Mb fine-mapped using the NMZ119/HHM population. The two red dashed lines in the middle represent the physical distance of 187.4 kb fine-mapped using the HHM/CS population. The sky-blue dots represent inversions between the genomes of Chinese Spring and other cultivars.

Due to differences in recombination rates between populations and issues with dominant markers (**Supplementary Figure S3**) between NMZ119 and HHM/CS, we suspect that there are differences in presence/absence variations (PAVs) in the *PmHHM* region between NMZ119 and HHM/CS. For conformation, we conducted a synteny analysis of the 740–754-Mb region among CS and 13 other common wheat genome sequences (**Figure 5**). As anticipated, a strong level of synteny was identified among Jagger, CDC Landmark, and CDC Stanley in the vicinity of *PmHHM*. However, the synteny between CS and the remaining 10 cultivars was disrupted due to significant sequence divergence in the specified region (**Figure 5**). These findings provide further support for our hypothesis that PAVs exist among different wheat varieties in the *PmHHM* gene region.

The targeted locus was mapped within an interval of 187.4 kb containing four high-confidence genes and seven low-confidence ones. Three of the high-confidence genes were of the NLR type (**Figure 3B**). Among the genomes analyzed, these genes were present in Jagger, CDC Landmark, CDC Stanley, and CS. However, they were missing in other genotypes, including Kenong 9204, Julius, Kariega, Renan, ArinaLrFor, LongReach Lancer, Mace, SY Mattis, Norin 61, and Zang 1817 (**Figure 3B**).

## Discussion

HHM displayed resistance to 25 *Bgt* isolates and was highly resistant in the field, making it a potentially valuable genetic resource for breeding programs. Here we showed that resistance in HHM was controlled by a single dominant gene located on chromosome arm 4AL. Fine-mapping of the resistance locus generated tightly linked molecular markers that should not only allow reliable selection of the locus in breeding programs but also provide a solid molecular platform for the positional cloning of *PmHHM*.

According to the physical location of the nine genes mentioned above that are mapped on wheat 4AL (**Figure 4**) and the allelic test results of this study, *PmHHM* is most likely allelic to *PmHHXM*, *PmXNM*, and *PmHSM*. Based on their responses to the 25 *Bgt* isolates, it is hypothesized that *PmHHM* differs from *PmHHXM* and *PmHSM*. However, we cannot determine whether there is a difference between *PmHHM* and *PmXNM*, as we are unable to obtain Xiaonanmai in this study. The precise relationship among these genes will be confirmed following the final cloning.

PAVs, a type of chromosomal structural variation found in plant genomes, are gaining more attention with the growing number of published wheat genome sequences. The presence of PAVs at the wheat pan-genome level is becoming more evident (Walkowiak et al., 2020; Zhou et al., 2021). On one hand, PAVs affect gene sequence variation and the functionality of neighboring genes associated with environmental stress and disease resistance in plants (Gabur et al., 2020; Lang et al., 2021; Zhai et al., 2021). On the other hand, PAVs could lead to incomplete chromosome pairing, resulting in a reduction in the recombination rate (Neu et al., 2002). The fact that the *PmHHM* region harbors PAVs among various common wheat accessions, combined with the amplification patterns of molecular markers between NMZ119 and HHM/CS (**Supplementary Figure S3**), indicates the presence of PAVs in the *PmHHM* gene region between NMZ119 and HHM/CS, leading to a decrease in the recombination rate in the *PmHHM* region of the NMZ119 population. Therefore, the selection of the correct susceptible parents is crucial during the fine-mapping of gene.

In the current study, we initially employed the large population of NMZ119/HHM to locate *PmHHM* within a physical span of 4.46Mb and identified 264 genes in this interval (data not shown). Among these genes, there are 102 resistance gene analogs, of which 47 are annotated as RPP13-like proteins 1. These resistance gene clusters may increase the likelihood of PAVs occurring at the gene locus, as the dynamic gene fraction, particularly enriched with disease resistance genes (Xue et al., 2021; Li et al., 2024a), is likely influenced by a continuous evolutionary arms race between plants and pathogens (Andersen et al., 2020). Subsequently, we ultimately mapped *PmHHM* within a 187.4-kb physical interval containing only three NLR-type genes using the large HHM/CS population. Our next steps will be (1) to isolate candidate genes from the target region and verify their functions using biotechnologies such as virus-induced gene silencing (VIGS), transgenesis, and gene editing and (2) to develop disease-resistant varieties using the closely linked markers for selection.

## Data Availability

The original contributions presented in the study are included in the article/**Supplementary Material**. Further inquiries can be directed to the corresponding author/s.
