# Supplementary material for: Fine-mapping of PmHHM, a broad-spectrum allele from a wheat landrace conferring both seedling and adult resistance to powdery mildew
Source: Front Plant Sci. 2025 Feb 6;15:1489013. doi: 10.3389/fpls.2024.1489013 (PMC11839664; doi:10.3389/fpls.2024.1489013)
Supplement: Supplementary file 2 [file Image1.pdf]

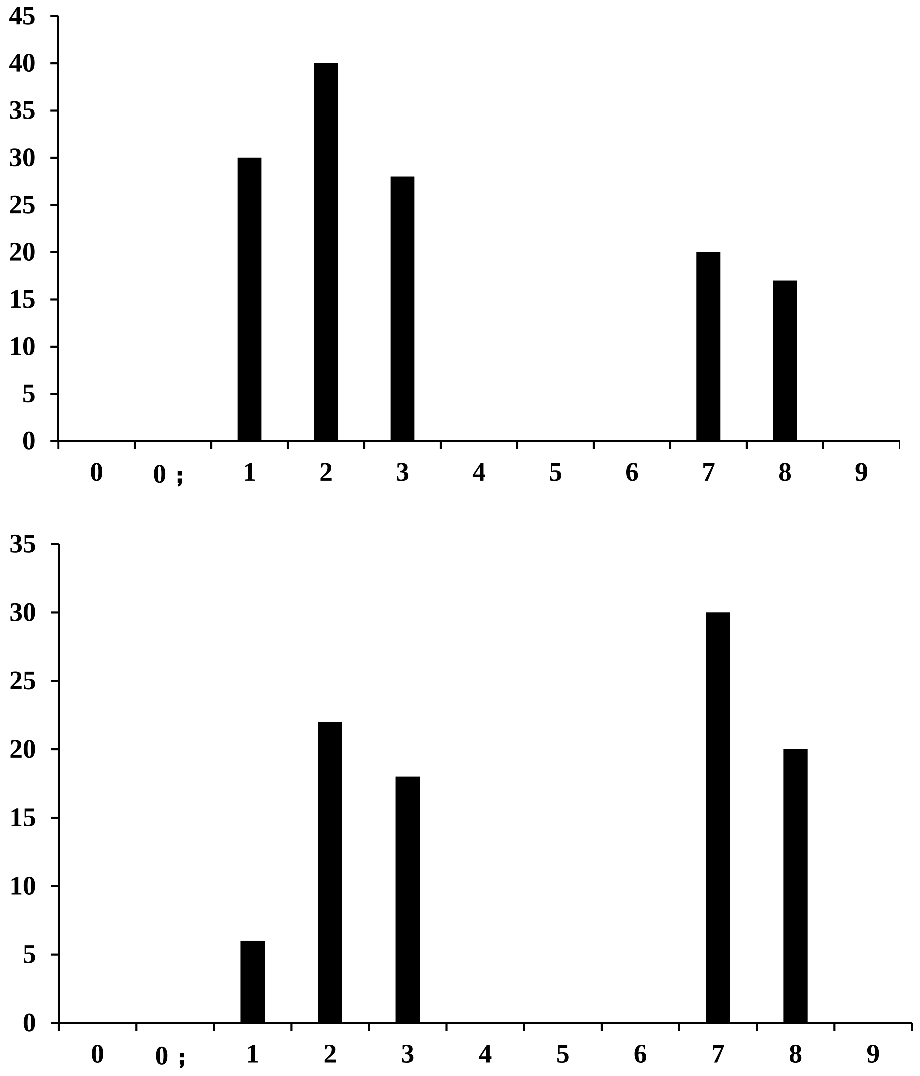

Fig. S1 Distribution of infection types in the BC_2F_2 (upper) and BC_3F_1 (lower) populations from NMZ119/HHM.

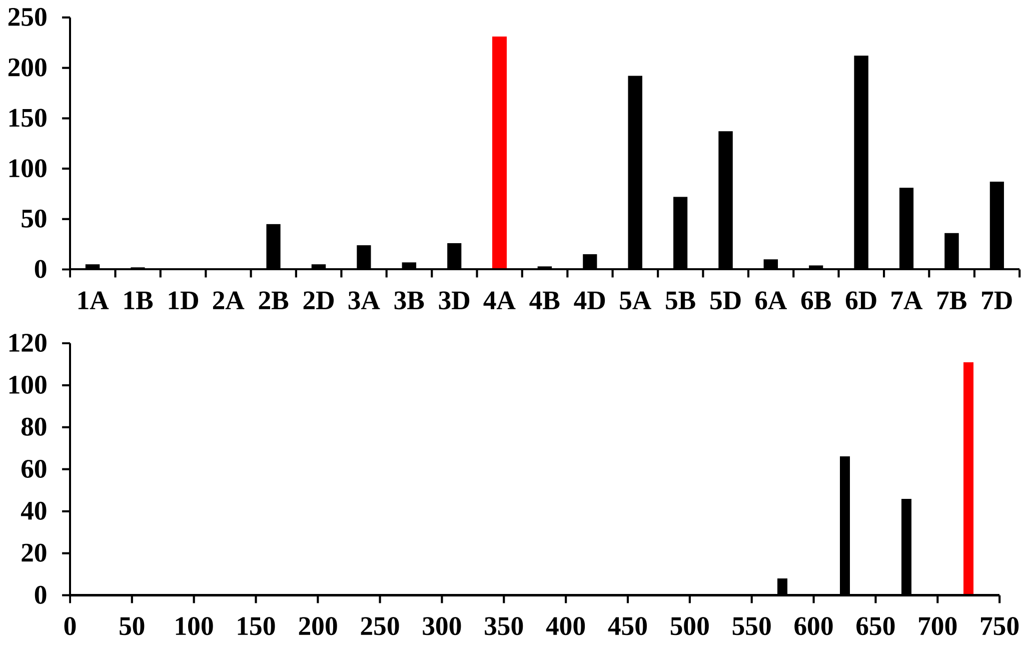

Fig. S2 Distribution of SNPs between the parents and between resistant and susceptible DNA bulks, respectively, across all 21 wheat chromosomes (upper), and within chromosome 4A (lower).

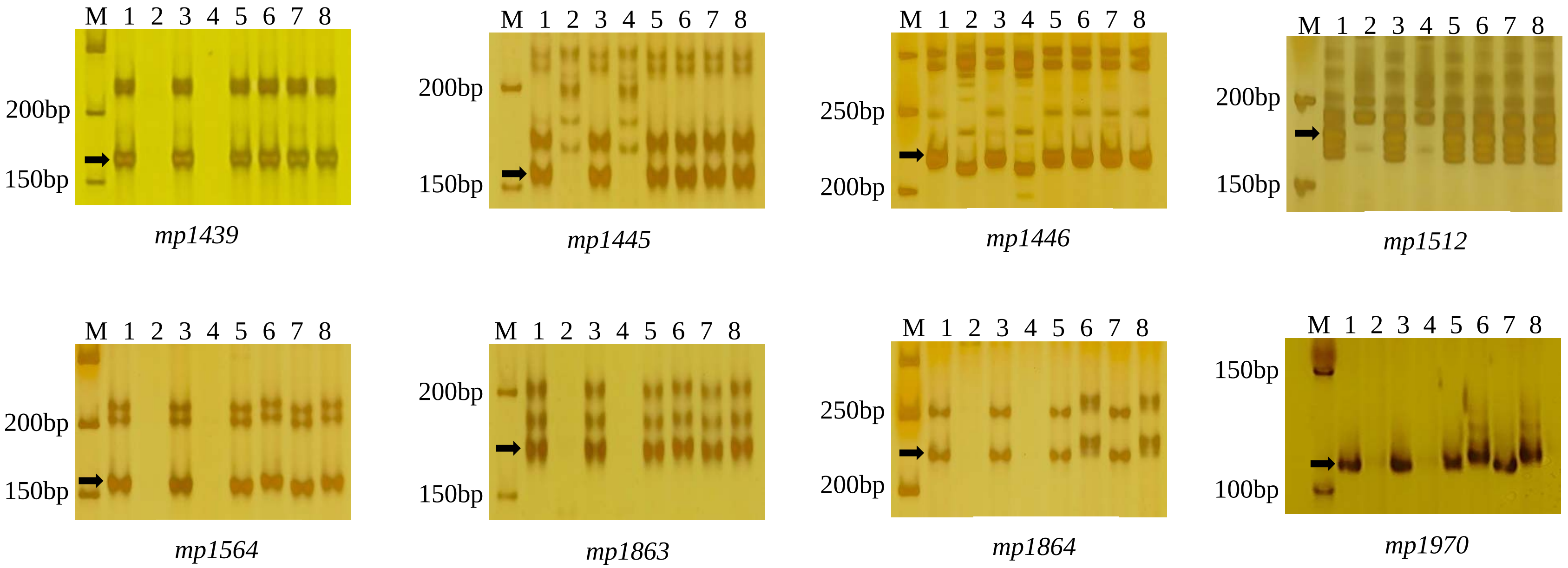

Fig. S3 PCR amplification of DNA from parents and bulks using eight marker primers on samples obtained. M: molecular weight standard; : 1, HHM; 2, NMZ119; 3, resistant pool; 4, susceptible pool; 5, HHM; 6, CS; 7, resistant pool; 8, susceptible pool.
